# Pulsatile Left Ventricular Assistance in High-Risk Percutaneous Coronary Interventions: Short-Term Outcomes

**DOI:** 10.3390/jcm13185357

**Published:** 2024-09-10

**Authors:** Josko Bulum, Marcelo B. Bastos, Ota Hlinomaz, Oren Malkin, Tomasz Pawlowski, Milan Dragula, Robert Gil

**Affiliations:** 1 Department of Internal Medicine, University Hospital Center Zagreb, 10000 Zagreb, Croatia; jbulum@kbc-zagreb.hr; 2 Thoraxcentrum, Erasmus Medical Center, 3015 GD Rotterdam, The Netherlands; 3 Department of Cardiology, International Clinical Research Center, St. Anne University Hospital and Masaryk, University School of Medicine, 656 91 Brno, Czech Republic; 4 PulseCath BV, 6811 KS Arnhem, The Netherlands; 5 Department of Cardiology, National Institute of Medicine, 02-507 Warsaw, Poland; 6 Department of Cardiology, University Hospital in Martin, 036 01 Martin, Slovakia; 7 Department of Cardiology, National Medical Institute of the Internal Affairs and Administration Ministry, 02-005 Warsaw, Poland

**Keywords:** registry, mechanical circulatory support, PulseCath, iVAC2L, pulsatile, high-risk, percutaneous coronary intervention, real world, LV assist device, coronary disease, heart failure, cardiogenic shock, ACS/NSTE-ACS, depressed LV function, mitral regurgitation dilated non-ischemic cardiomyopathy, ischemic cardiomyopathy

## Abstract

Objectives: To document the real-world experience with the use of pneumatic pulsatile mechanical circulatory support (MCS) with the PulseCath iVAC2L during high-risk percutaneous coronary interventions (HR-PCIs). **Background**: The use of MCS in HR-PCIs may reduce the rate of major adverse cardiovascular events (MACEs) at 90 days. The PulseCath iVAC2L is a short-term pulsatile transaortic left ventricular (LV) assist device that has been in use since 2014. The iVAC2L Registry tracks its safety and efficacy in a variety of hospitals worldwide. **Methods**: The iVAC2L Registry is a multicenter, observational registry that aggregates clinical data from patients treated with the iVAC2L worldwide. A total of 293 consecutive cases were retrospectively collected and analyzed. Estimated rates of in-hospital clinical endpoints were described. All-cause mortality was used as the primary endpoint and other outcomes of interest were used as secondary endpoints. The rates obtained were reported and contextualized. **Results**: The in-hospital rate of all-cause mortality was 1.0%, MACE was 3.1%. Severe hypotension occurred in 8.9% of patients. Major bleeding and major vascular complications occurred in 1.0% and 2.1%, respectively. Acute myocardial infarction occurred in 0.7% of patients. Cerebrovascular events occurred in 1.4% of patients. Cardiac arrest occurred in 1.7% of patients. A statistically significant improvement in blood pressure was observed with iVAC2L activation. **Conclusions**: The results of the present study suggest that the iVAC2L is capable of improving hemodynamics with a low rate of adverse events. However, confirmatory studies are needed to validate these findings.

## 1. Introduction

Due to the recent advances in cardiovascular therapeutics, the growing use of short-term mechanical circulatory support (MCS) devices is now an established trend. MCS is available in a variety of support modalities, each with the common goal of improving prognosis after high-risk invasive interventions.

The intra-aortic balloon pump (IABP) is the most commonly utilized modality; however, its use tends to decrease in favor of new and more powerful ones, which now have class IIb recommendation for prophylactic use in high-risk percutaneous coronary interventions (HR-PCIs) [1,2,3]. Despite evidence favoring pulsatile flow [4,5], all current short-term MCS devices are continuous-flow devices (CFDs), with PulseCath iVAC2L (PulseCath BV, Arnhem, The Netherlands) being the only exception. Numerous large-scale studies have examined the utilization of short-term MCS in a real-world context. Nevertheless, to date, no reports have addressed the use of pulsatile flow (PF) in a real-world setting.

Three single-center prospective studies have already demonstrated the safety and efficacy of the iVAC2L [5]. The iVAC2L Registry is an open, multicenter registry that collects data on the real-world use of the iVAC2L on a global scale. This report presents the initial findings from the iVAC2L Registry.

## 2. Methods

**Description of the iVAC2L.** The iVAC2L comprises a membrane pump connected to a nitinol-reinforced polyurethane bi-directional flow catheter. The catheter has a length of 92 cm, with an outer diameter of 17 French (Fr). It features a stainless steel inlet tip and a patented two-way valve, illustrated in Supplementary Figure S1. The chamber is subdivided by a flexible membrane into a blood chamber and a helium chamber. The catheter is then inserted into the femoral artery and positioned with the inlet tip inside the LV, where it operates in synchrony with the cardiac cycle. During diastole, the helium chamber receives helium from the IABP driver, resulting in the ejection of blood back into the ascending aorta with a stroke volume of around 21 mL. During systole, the helium chamber undergoes a deflation, which is accompanied by a refill of the blood chamber. The pump typically produces an additional flow of 1.5 to 1.8 L/min, though it has the capacity to reach up to 2.5 L/min (data not yet published). The optimal performance of the device is achieved at rates between 70 and 90 beats per minute (bpm).

**Study population and data collection:** The iVAC2L Registry is a multicenter, international registry that retrospectively collects data on the utilization of iVAC2L in a range of indications. Participation is entirely voluntary. In order to guarantee strict adherence to the Declaration of Helsinki, it is recommended that operators seek verbal consent from patients regarding their willingness to have their data utilized for research purposes and to provide data only upon confirmation.

To ensure the highest degree of completeness and accuracy, data were collected via the use of a standardized Clinical Report Form (CRF). Furthermore, all information was completed at the site with the assistance of the clinical team. The data sources included medical records, ancillary diagnostic tests, specialist reports, and communications from treating physicians. Subsequently, the CRFs were subjected to a review by a medical specialist for coherence, completeness, and accuracy.

The present analysis encompasses individuals aged 18 years and above who underwent iVAC2L during HR-PCIs for coronary artery disease, and excludes procedures driven by indications other than HR-PCIs, as well as cases of acute extra-cardiac disease. The data were anonymized at the time of collection and remained anonymous throughout the analysis. HR-PCI was defined as any percutaneous intervention for coronary disease involving an unusually high risk of periprocedural circulatory collapse, as determined by the treating physician.

**Clinical Endpoints.** The data were collected retrospectively. The adjudication of events was conducted by the attending physicians in accordance with the prevailing standards of practice at the local level. The primary endpoint was all-cause in-hospital mortality. Secondary endpoints included major adverse cardiovascular events (MACEs), major vascular complications, major bleeding, acute kidney injury (AKI), and the occurrence of severe hypotension while on circulatory support. The composite endpoint of MACE was defined as the occurrence of in-hospital all-cause mortality, cerebrovascular event, acute myocardial infarction (AMI), and repeat revascularization. Severe hypotension was defined as a mean arterial pressure (MAP) of less than 60 mmHg for a minimum of 10 min or the presence of shock of any etiology. A major vascular complication was defined as a clinically significant vascular injury, bleeding, or limb ischemia. All other endpoints were adjudicated at the local level by local teams using local standards of practice and definitions.

**Statistical Analysis.** Continuous data are presented as either mean ± SD or median (25th–75th quartiles), as appropriate. Categorical data are presented as frequencies and percentages. The results of the study include a description of the subjects’ baseline characteristics, procedural data, and clinical endpoints. Baseline and procedural data are presented for descriptive purposes, and no statistical tests were performed. The hemodynamic data were analyzed utilizing either Student’s *t*-test for paired samples or the Wilcoxon signed-rank test for paired samples, depending on the statistical distribution. The Bonferroni correction was employed to account for multiplicity. To investigate the major factors associated with severe hypotension and in-hospital MACE, uni- and multivariate logistic regressions were conducted. For the multivariate analyses, stepwise backward and forward selections were employed. The data were collected and stored using Microsoft Excel 2019 (Microsoft Corporation, Redmond, WA, USA), and the analysis was performed using the R v4.0.2 statistical package. All statistical tests were based on a two-tailed significance level of 5%.

## 3. Results

**Figure 1:**
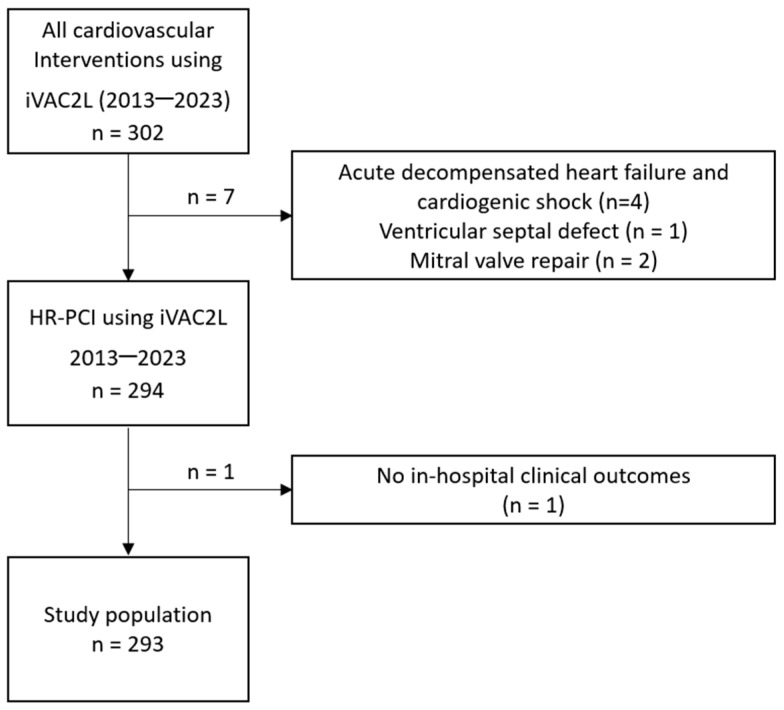
Study flowchart. HR-PCI: high-risk percutaneous coronary intervention.

**Figure 2:**
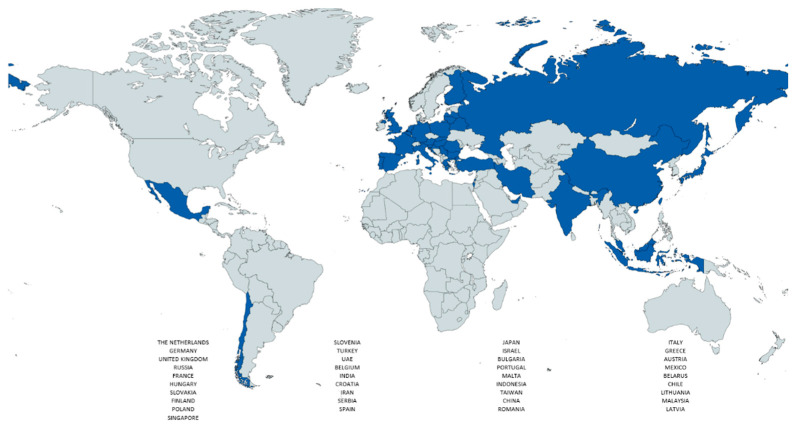
Countries whose centers provided data to the iVAC2L Registry until 2023 (in blue).

A total of 302 consecutive patients received iVAC2L during high-risk cardiovascular procedures in 37 countries between November 2013 and October 2023 (Figure 1 and Figure 2). Of the aforementioned patients, seven were excluded from the analysis due to indications other than HR-PCI, and one due to the lack of clinical outcomes. In-hospital outcomes were ascertainable in 99.7% of cases (*n* = 293).

The baseline demographic and procedural characteristics are presented in Table 1; Table 2, respectively. The median age was 71 years (range: 64–77 years), and 85% of the participants were male. The ejection fraction (EF) was 30% (25–40%), and the SYNTAX score was 33 (28–40). The majority of patients exhibited moderate to severe symptoms of heart failure, with 71% classified as NYHA class III or IV. Additionally, 50% of the cohort was deemed ineligible for CABG. A history of acute myocardial infarction (AMI) was present in 53% of the cohort, while renal insufficiency, previous cerebrovascular events, and peripheral arterial disease were also common.

**Procedural characteristics.** Lesions in the left main coronary artery or the left anterior descending artery and its branches were treated in 59% and 71% of cases, respectively. The median duration of the procedure was 67 (45–100) minutes. Rotational atherectomy was employed in 19% of cases. Data on the maximum flow produced by the iVAC2L were available in 63% of cases and were estimated to be 1.6 (1.4–1.7) L/min, with a range of (1.0–2.5) L/min. The MAP at baseline was 80 ± 17 mmHg, with a systolic blood pressure (SBP) of 117 ± 24 mmHg.

**Figure 3:**
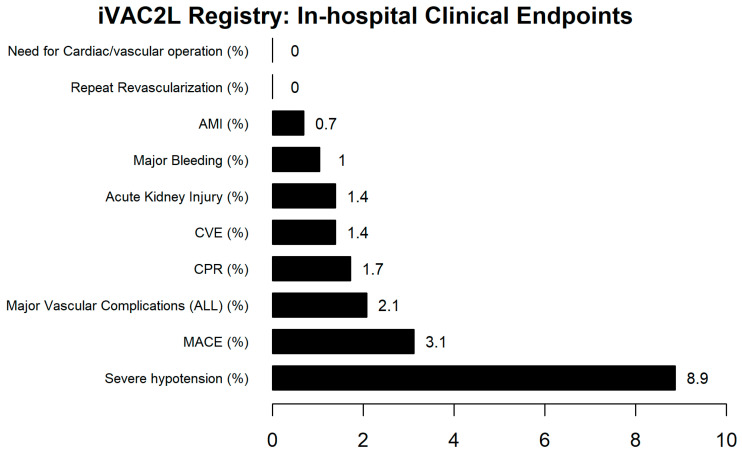
In-hospital clinical outcomes from the iVAC2L Registry. MACE: Major Adverse Cardiovascular Event. AMI: Acute Myocardial Infarction. CVE: Cerebrovascular Event. CPR: Cardiopulmonary Resuscitation.

**Figure 4:**
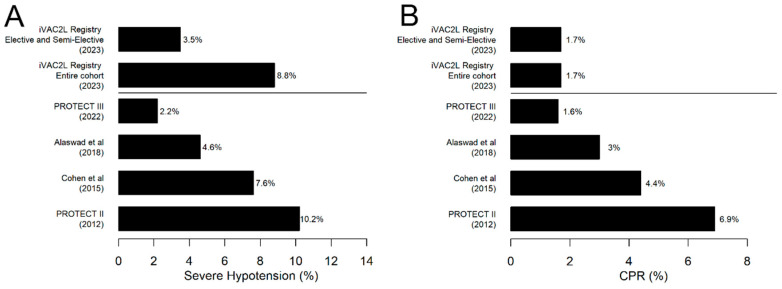
(**A**) Rates of severe hypotension found in the most relevant studies on the use of short-term mechanical circulatory support in high-risk PCI. (**B**) Rates of CPR found in the most relevant studies on the use of short-term mechanical circulatory support in high-risk PCI [3,6,7]. CPR: Cardiopulmonary Resuscitation.

**Clinical Endpoints.** A summary of the clinical endpoints is provided in Figure 3 and Figure 4. The incidence of all-cause mortality, cardiovascular events (CVEs), and acute myocardial infarction (AMI) was 1.0%, 1.4%, and 0.7%, respectively. A total of 3.1% of cases resulted in a MACE. Major vascular complications were observed in 2.1% of cases, none of which necessitated urgent cardiac or vascular surgery. Major bleeding was observed in 1.0% of cases. One patient presented with a major bleeding complication that required surgical intervention but succumbed before surgery could be performed. Dislodgement of the iVAC2L occurred in 5% of cases, while the necessity for removal of the iVAC2L (including escalation to alternative devices) was observed in 1.0% of instances. Endotracheal intubation was required in 3.7% of cases. Severe hypotension occurred in 8.9% of cases. Cardio-pulmonary resuscitation (CPR) was necessary in 1.6% of cases. When only elective or semi-elective procedures were selected (*n* = 174), these rates were 3.5% and 1.7%, respectively.

**Figure 5:**
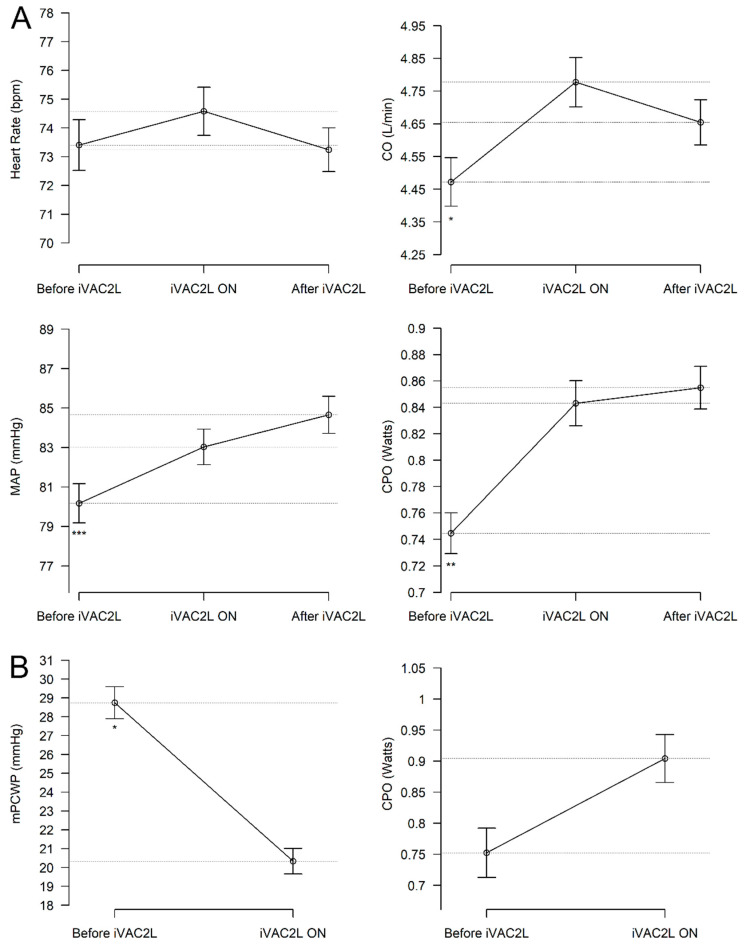
(**A**) Hemodynamic changes obtained with the activation of iVAC2L showing improvements in blood pressure, CO, and CPO. (**B**) Hemodynamic measurements taken before implementation of iVAC2L and during support. Data are presented as mean ± SE. *p*-values derive from t-tests or Wilcoxon’s test for paired samples. * *p* < 0.05; ** *p* < 0.01; *** *p* < 0.01 compared to “iVAC 2L ON”. CO: cardiac output. MAP: mean arterial pressure. CPO: cardiac power output. mPCWP: mean pulmonary capillary wedge pressure.

**Hemodynamic effects.** Findings are shown in Figure 5 and in Supplementary Table S1. Data on heart rate, blood pressure, cardiac output and mPCWP were available in 78%, 76%, 17%, and 17% of all cases, respectively.

Relative to the pre-support state (i.e., baseline), measurements taken with iVAC2L activated showed significant increases in both SBP (117.3, 95%CI: [114.2–120.4] vs. 121.2, 95%CI: [118.2–124.2] mmHg, *p* < 0.01) and DBP (61.3, 95%CI: [59.2–63.5] vs. 63.8, 95%CI: [61.9–65.8] mmHg, *p* < 0.01). The heart rate remained constant (73.4, 95% CI: [71.4–75.4] vs. 74.6, 95% CI: [72.7–76.4] bpm, *p* = 0.26). There was a significant increase in CO (4.47, 95% CI: [4.13–4.81] vs. 4.78, 95% CI: [4.42–5.14] L/min, *p* < 0.05) and CPO (0.74, 95% CI: [0.67–0.82] vs. 0.84, 95% CI: [0.76–0.92] Watts, *p* < 0.01). No alterations were observed in the mPCWP (15.8, 95% CI: [13.6–18.1] vs. 17.1, 95% CI: [14.7–19.6] mmHg, *p* = 0.14) when assessed in the entire cohort.

In patients who received inotropes and/or vasopressors at any point (*n* = 27, 9.2%), cardiac output (CO), blood pressure, and heart rate remained unaltered. However, CPO increased numerically in this subgroup from 0.75 (95% CI: [0.52, 0.99]) vs. 0.9 (95% CI: [0.68, 1.13]) Watts, *p* = 0.37. Additionally, mPCWP demonstrated a non-significant trend toward a decrease from 28.8 (95% CI: [24.4, 33.1]) vs. 20.3 (95% CI: 16.4, 24.3) mmHg, *p* = 0.08.

**Figure 6:**
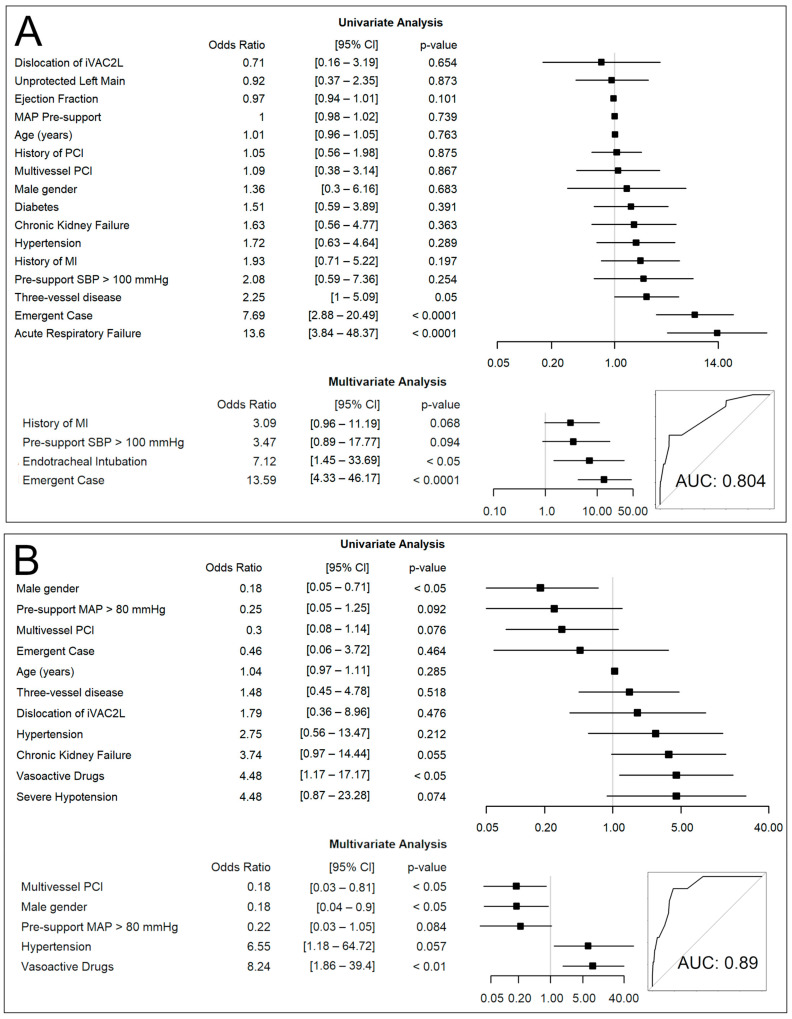
Uni- and multivariate logistic regression analysis: (**A**) predictors of intraprocedural severe hypotension; (**B**) predictors of in-hospital MACE; MACE: Major Adverse Cardiovascular Event. MAP: Mean Arterial Pressure; AUC: Area Under the Curve; PCI: Percutaneous Coronary Intervention.

Independent predictors of severe hypotension were Emergent Case and Endotracheal Intubation. Previous MI and a pre-procedural SBP > 100 mmHg were non-significantly related (AUC: 0.80). Independent predictors of in-hospital MACE were Multivessel PCI, Male Gender, Pre-support MAP > 80 mmHg, Hypertension, and Vasoactive Drugs, AUC = 0.89 (Figure 6).

## 4. Discussion

This study represents the most comprehensive compilation of clinical data on HR-PCI using PulseCath iVAC2L since the device received CE Mark approval in 2014. The in-hospital mortality rate was 1%, and the MACE rate was 3.1%. The findings also demonstrate beneficial effects on the systemic circulation, and the large sample size lends representativeness to real-world practice.

### 4.1. Previous Evidence on the iVAC2L

Since 2015, three prospective cohorts have been published, all in the context of elective HR-PCIs. Den Uil et al. reported hemodynamic improvements in elective HR-PCIs with no major adverse events. Samol et al. described that both iVAC2L and Impella 2.5 significantly increased the MAP, but Impella 2.5 precipitated hemolysis. The PULSE trial showed a 14% reduction in the metabolic demands, a 33% reduction in the native heart’s cardiac output, and a 17% increase in the MAP [5]. The iVAC2L may be an alternative when there is a need for an LVAD that is less associated with hemolysis than a CFD or when there is great concern regarding the possibility of iatrogenic valvular damage that could precipitate circulatory collapse [8]. It may also be a more feasible alternative in hospitals where other percutaneous MCS devices are not yet reimbursed.

### 4.2. Study Findings

The output flow of the iVAC2L ranged between 1.0 and 2.5 L/min. This can be up to four times that of the IABP, approaching the output flow of Impella 2.5 (1.9 ± 0.27 L/min). It remains lower than that of the Impella CP (2.8 ± 0.4 L/min at P8), though [5,6,9].

The intraprocedural incidence of severe hypotension was 8.9%, which is lower than the rate observed in the Impella arm of the PROTECT II trial (10.2%) [3]. The PROTECT II evaluated the effects of Impella 2.5 versus IABP in elective HR-PCIs and remains the largest RCT in this population to date. As with the PROTECT II study, the iVAC2L Registry comprised a high-risk cohort with low EF, a high SYNTAX score, a history of frequent revascularization and/or AMI, and a high frequency of rotational atherectomy. It should be noted, however, that the registry also includes cases requiring emergent intervention. The odds of developing intraprocedural severe hypotension were 14 times higher in emergent cases compared to non-emergent cases. This parameter was identified as the strongest independent predictor of severe hypotension (Figure 6). In non-emergent cases, the incidence of severe hypotension was 3.5%, which is lower than that observed in elective and semi-elective studies using Impella, namely 7.1% (2015) and 4.6% (2018) [6,7]. While a 2.2% rate was recently reported in 2022 by O’Neill et al., it should be noted that patients in this study were healthier, with a SYNTAX score of 28 versus 31 in PROTECT II and 33 in the iVAC2L Registry. Our findings corroborate those of previous studies which have demonstrated that planned MCS is associated with less hemodynamic instability, which in turn is associated with lower odds of MACEs. Consonantly, previous data shows that non-emergent implantation leads to reduced rates of mortality, CPR, and lesser need for inotropes/vasopressors [10,11].

Activation of iVAC2L resulted in significant increases in MAP, SBP, DBP, CO, and CPO (Figure 5A). mPCWP decreased numerically with the activation of iVAC2L, but only in those receiving vasoactive medications during support. The aforementioned medications, which include vasoactive drugs such as inotropes and vasopressors, can be employed as surrogates for critical decompensated states in the majority of cases (Figure 5B). The observed changes are consistent with those reported in previous studies on the same device. Of particular clinical interest is the observed increase in CPO, as a CPO value below 0.6 Watts has been associated with worse clinical outcomes [12]. However, in the current study, CPO was only reported in 17% of cases, and thus it should be interpreted with caution.

The results demonstrate a reduction in the incidence of CPR episodes with iVAC2L (1.7%) in comparison to the Impella arm of PROTECT II (6.9%). This figure is also lower than the 3% reported by Alaswad et al. for the Impella device. However, it is comparable to the 1.6% reported in the PROTECT III report. Unlike Alaswad et al., PROTECT III predominantly used Impella CP rather than 2.5, suggesting equivalent performance between iVAC2L and Impella CP [3,7]. Although a reduction in mPCWP could only be detected in a critical state, only 3.7% of the total cohort required advanced airways compared to 5% with (mostly) CFDs in a previous study [5]. This may indicate that pulsatile flow (PF) facilitates improved unloading, although the observational design precludes the drawing of causal inferences.

The in-hospital mortality rate following mechanically assisted HR-PCI ranges from 3.2% to 11.5% [13]. The PROTECT II and the cVAD Registry reported in-hospital mortality rates of 4.6% and 3.3%, respectively [3,7]. In contrast, our findings indicate a 1% rate with iVAC2L, which is comparable to the 0.7% observed in the EUROPELLA registry [14]. The two studies exhibited similarities with regard to the frequency of left main stenting, history of CABG, and age distribution. However, the EUROPELLA excluded emergent cases (Supplementary Table S2). Recently, two studies have reported even higher rates of 7.4% with Impella CP and 14% with VA-ECMO, but they had small sample sizes and more complex interventions. While this compromises comparability with larger studies, the rate observed with ECMO is not unexpected, since this modality has been shown to increase mortality, vascular complications, and hemolysis [15,16,17].

The current analysis reveals a 3.1% incidence of in-hospital MACE which is less than the rates observed in the PROTECT II, PROTECT III, and the cVAD registry (10.2%, 5.4%, and 4.3%, respectively). This may be attributed to the utilization of PF, but also to the technical advancements since 2012. Although the PROTECT III was recently published, it employed rotational atherectomy more frequently, which might provide an explanation to the higher rate of MACE in comparison to iVAC2L [3,9]. Nevertheless, in the iVAC2L Registry, MACE was not determined by rotational atherectomy, but rather by the administration of vasoactive drugs, which are indicative of hemodynamic instability. As in clinical practice, the latter was primarily influenced by the urgency of the intervention. Another predictor of interest was multivessel PCI, a finding that has also been observed in large-scale studies where a more complete revascularization has been associated with superior outcomes. This finding suggests that the benefit observed in the FAME-2 and PROTECT II studies [3,18] at longer time frames may also be applicable in the short term. Moreover, this serves to reinforce the significance of the iVAC2L Registry as a reflection of contemporary clinical practice.

With the exception of CVE, all other clinical outcomes compared favorably with previous large studies (Supplementary Figure S2). Four patients had acute neurological deficits during or after the procedures. Notwithstanding, the observed incidence of stroke is lower than that expected for Impella, which is approximately 2.6% [13].

The use of PF instead of CF may be advantageous for several reasons. CF may reduce or eliminate aortic leaflet motion and coaptation, increasing the risk of acute thrombosis in the ascending aorta. It also decreases sensitivity to catecholamines in the peripheral vasculature and is reported to be less effective in maintaining end-organ perfusion while increasing aortic impedance and systemic vascular resistance [19]. The mechanisms involved include stiffening of the arterial system with obliteration of the Windkessel effect and increased backward propagation of reflected waves. This elevates the afterload and may jeopardize the unloading effect [4,12].

Counter-pulsation as applied by iVAC2L is probably less detrimental to LV afterload and more effective in unloading the LV chamber, resulting in more pronounced reductions in the myocardial metabolic demands. Previous reports have also documented significant reductions in intraventricular dyssynchrony [5,20]. In the peripheral circulation, PF can facilitate blood flow to end organs. It restores cyclic stress and delivers a greater amount of energy to the walls of peripheral vessels in comparison to CF. This has been linked to a reduction in postoperative mortality [4,19,20,21]. The evidence indicates that PF is more beneficial than CF for vital organ perfusion, as evidenced by studies on the stomach, liver, and kidney [22]. Consonantly, the levels of severe hypotension found in this analysis were remarkably low.

The aforementioned capabilities enhance the resilience of the cardiovascular system to ischemic injury, reducing hemodynamic instability, and consequently facilitating more extensive and meticulous interventions. The RESTORE-EF, PROTECT II, and Roma-Verona studies have demonstrated that patients who received a more complete revascularization exhibited greater improvements in LVEF after 90 days [3,23,24]. Concurrently, a meta-analysis including more than 17,000 individuals indicated that the utilization of intravascular imaging enhanced patient outcomes [25]. Furthermore, the augmented stability provided by MCS permits the broader deployment of hyperemic agents to evaluate lesion significance in patients with markedly impaired LV function, consequently reducing the incidence of repeat interventions [18].

## 5. Merits and Flaws

The present analysis includes a wide range of hospitals around the world and sheds light on the real-world use of iVAC2L in HR-PCI with focus on safety and efficacy. As in other relevant studies, the study population is characterized by a high-risk profile. The results also demonstrate a high prevalence of left main stenting and rotational atherectomy. Nevertheless, the observational design entails a risk of bias due to its potential for selective reporting, which precludes definitive conclusions on causality. Moreover, despite the lack of consistent follow-up beyond the immediate postoperative period, the initial hours were comprehensively documented. The majority of adverse events (approximately 70%) tend to occur within the first hours during and immediately after the PCI [26]. Therefore, while some limitations in follow-up may influenced the observed rates, this effect is most likely minor in magnitude.

It is not possible to discount the possibility that unmeasured confounders, such as the administration of intravenous fluids or the inherent effects of revascularization, may have influenced the results. Nevertheless, it can be argued that the observed hemodynamic response is consistent with that reported in previous studies [5]. Although arterial blood pressure data were widely available, pulmonary monitoring occurred in only 17% of cases, which limits the generalizability of the findings and reduces the study’s power to draw definitive conclusions on the variations observed in CO and CPO. Additionally, the limited availability of angio-CT may have influenced the incidence of vascular complications. In light of these considerations, the present analysis should be regarded as hypothesis-generating only.

## 6. Future Research

Future research should incorporate more structured designs, such as matched cohorts, to address the limitations of the observational design. This should include the adoption of a prospective design, the incorporation of a clinical events committee, written informed consent, long-term follow-up for up to one year, and electronic centralized data collection. Hemodynamic evaluations should include details on timing relative to stenting, vasoactive drugs, intravenous fluids, and other confounders. In the coming years, the UNLOAD-CHIP trial will measure 30-day mortality or cardiogenic shock in 98 stable high-risk patients with low EF randomized to iVAC2L versus no MCS. The PULSE II trial will assess major adverse events (MAEs) at 90 days in 368 elective high-risk subjects randomized to iVAC2L (PF) or Impella CP (CF). In China, an RCT will investigate the incidence of MACEs at 30 days in 316 stable high-risk patients randomized to iVAC2L or IABP. By 2026, the PROTECT IV (NCT04763200) trial, which compares Impella CP with no MCS, will provide further evidence on CFDs. Additional gaps in knowledge include the impact of intermittent superior vena cava occlusion on the effects of iVAC2L and the impact of iVAC2L on the need for vasoactive drugs.

## 7. Conclusions

The PulseCath iVAC2L registry demonstrates low rates of in-hospital mortality and MACEs with the use of pulsatile MCS in a real-world setting. This demonstrates its potential to be a safe and effective tool to improve clinical outcomes after complex coronary interventions.

## Figures and Tables

**Table 1:** Baseline demographics, *n* = 293. Data are presented as mean ± SD or median (25th to 75th quartiles) as appropriate. Count data are presented as percentages (no. of available observations).

Baseline Characteristics	
Age (years)	71 (64 to 77)
SYNTAX	33 (28 to 40)
EF (%)	30 (25 to 40)
Gender (male) (%)	84.8 (264)
NYHA classification III/IV (%)	71.3 (174)
EF < 40% (%)	69.2 (273)
Hypertension (%)	76.4 (216)
Type II Diabetes (%)	38.5 (218)
Previous Stroke (%)	9.3 (205)
Previous Myocardial Infarction (%)	52.5 (217)
Peripheral Artery Disease (%)	13.9 (216)
Renal Insufficiency (%)	23.3 (219)
Cancer (%)	4.5 (176)
Not surgical candidate (%)	50 (224)
Previous PCI (%)	38.5 (208)
Previous CABG (%)	33.7 (208)
Three-vessel Disease (%)	56.1 (278)
Unprotected Left Main (%)	35.1 (228)
Multivessel Disease (%)	72.3 (285)

**Table 2:** Procedural characteristics, *n* = 293. Data are presented as mean ± SD or median (25th to 75th quartiles) as appropriate. Count data are presented as percentages (no. available observations).

Procedural Characteristics	
Stented LM (%)	59.1 (274)
Stented LAD and branches (%)	70 (249)
Stented LCX and branches (%)	50.8 (252)
Stented RCA and branches (%)	31.9 (254)
Heart Rate (bpm)	71 (63.8 to 80)
SBP (mmHg)	117.3 ± 23.7
DBP (mmHg)	61.3 ± 16.3
MAP (mmHg)	80 ± 16.8
CPO (Watt)	0.75 ± 0.27
CO (L/min)	4.47 ± 1.27
Pump flow (L/min)	1.6 (1.4 to 1.7)
Rotational Atherectomy (%)	19 (183)
Support time (min)	67 (45 to 100)

## Data Availability

Restrictions apply to the availability of these data. Data were obtained from PulseCath BV and are available from the authors with the permission of PulseCath BV.

## Supplementary Materials

The following supporting information can be downloaded at: https://www.mdpi.com/article/10.3390/jcm13185357/s1, Figure S1: The iVAC2L concept; Figure S2: Rates of in-hospital clinical outcomes; Table S1: Rates of Adverse Events; Table S2: Baseline data; Table S3: Hemodynamic measurements.

## Abbreviations

AKI	Acute kidney injury
AMI	Acute myocardial infarction
CABG	Cardio-pulmonary bypass graft
CAD	Coronary artery disease
CO	Cardiac output
CF	Continuous flow
CFD	Continuous flow device
CPO	Cardiac power output
CVE	Cerebrovascular event
DBP	Diastolic blood pressure
EF	Ejection fraction
HR-PCI	High-risk percutaneous coronary intervention
IABP	Intra-aortic balloon pump
IQR	Interquartile range
LM	Left main
LV	Left ventricle
LVAD	LV assist device
MACE	Major adverse cardiovascular events
MAP	Mean arterial pressure
MCS	Mechanical circulatory support
mPCWP	Mean pulmonary artery wedge pressure
PCI	Percutaneous coronary intervention
PF	Pulsatile flow
RCT	Randomized controlled trial
SBP	Systolic blood pressure
VA-ECMO	Veno-arterial extra-corporeal membrane oxygenation
