# Supplementary material for: Pulsatile Left Ventricular Assistance in High-Risk Percutaneous Coronary Interventions: Short-Term Outcomes
Source: J Clin Med. 2024 Sep 10;13(18):5357. doi: 10.3390/jcm13185357 (PMC11432136; doi:10.3390/jcm13185357)
Supplement: Supplementary file 1 [file jcm-13-05357-s001.zip › jcm-3113677-supplementary.pdf]

Pulsatile Left Ventricular Assistance in High-Risk Percutaneous Coronary Interventions: Short-Term Outcomes

Josko Bulum, Marcelo B. Bastos, Ota Hlinomaz, Oren Malkin, Tomasz Pawlowski, Milan Dragula and Robert Gil

Supplement

STUDY	EUROPELLA	PROTECT II	USPELLA	USPELLA 2015	PROTECT III	IVAC2L REGISTRY
YEAR	2009	2012	2012	2015	2022	2023
REFERENCE NUMBER						
N	144	175	504	637	216	293
AGE (years)	71.8±9.9	67.5±11	70±10	70.2±11.5	69.3±11.2	71 (64 - 77)
SYNTAX	---	30.5±13.2	37±16	---	28±12.6	33 (28 - 40)
EJECTION FRACTION (%)	---	23.4±6.3	31±17	30.1±16.1	22.6±7.1	30 (25 - 40)
GENDER (MALE) (%)	81.3	80.6	74	73.5	76.2	85 (224)
NYHA CLASSIFICATION III/IV (%)	---	67.4	66	70.3	75.9	71 (124)
HYPERTENSION (%)	67.4	88	---	---	88.3	76 (165)
TYPE II DIABETES (%)	43.1	53.2	47	50.5	55.8	39 (84)
PREVIOUS STROKE (%)	13.9	12	22	9.7	15.4	9 (19)
PREVIOUS AMI (%)	52.8	69.3	56	51.6	40.7	53 (114)
PERIPHERAL ARTERY DISEASE (%)	22.2	---	---	30.2	---	14 (30)
RENAL INSUFFICIENCY (%)	28.5	22.7	33	31.3	26.1	23 (51)
CANCER (%)	---	---	---	---	---	5 (8)
NOT SURGICAL CANDIDATE (%)	43.1	63.4	56	14	57	50 (112)
PREVIOUS PCI (%)	---	---	48	46.6	---	38 (80)
PREVIOUS CABG (%)	29.2	39.4	28	29.6	13.7	34 (70)
THREE-VESSEL DISEASE (%)	---	14.4	---	10.1	31.4	56 (156)
MULTIVESSEL DISEASE (%)	81.9	---	89	71.6	---	72 (206)
STENTED LM (%)	52.8	28.2	---	16.1	43.3	59 (162)
STENTED LAD AND BRANCHES (%)	---	58.8	---	33.2	75.2	70 (175)
STENTED LCX AND BRANCHES (%)	---	53.2	---	29.3	62.2	51 (128)
STENTED RCA AND BRANCHES (%)	---	34.6	---	17.1	29.2	32 (81)
NUMBER OF STENTS DEPLOYED	---	---	2±1.1	2.21±1.14	---	2 (1.2 - 3)
SBP (mmHg)	130±26.2	---	119±25	120±24	---	117.3±23.7
DBP (mmHg)	66.8±15.4	---	64±15	68±14	---	61.3±16.3
MAP (mmHg)	---	88.4±14.6	83±18	86±16	88.2±15	80±16.8
CPO (Watts)	---	---	---	---	---	0.7±0.3
CO (L/min)	---	---	---	---	---	4.5±1.3
PUMP FLOW (L/min)	---	---	2.1±0.2	---	---	1.6 (1.4 - 1.7)
ROTATIONAL ATHERECTOMY (%)	---	14.8	15	18.1	37.1	18 (33)
SUPPORT TIME (min)	87.8±50.7	81±44.4	60±53	134.4±371.4	99±62.4	67 (45 - 100)
NUMBER OF VESSELS TREATED	---	1.8±0.7	---	1.82±0.59	2.1±0.7	2 (1 - 3)

Supplementary Table S2. Baseline demographics and procedural characteristics of the iVAC2L Registry and similar data reported in other relevant studies performed with transaortic devices. AMI: Acute Myocardial Infarction. CABG: Coronary Artery Bypass Graft. CO: Cardiac Output. CPO: Cardiac Power Output. DBP: Diastolic Blood Pressure. LAD: Left Anterior Descending. LCX: Left Circumflex. LM: Left Main. MAP: Mean Arterial Pressure. PCI: Percutaneous Coronary Intervention. RCA: Right Coronary Artery. SBP: Systolic Blood Pressure.

IN-HOSPITAL CLINICAL ENDPOINTS	%
ALL-CAUSES MORTALITY (%)	1.0
CVE (%)	1.4
AMI (%)	0.7
REPEAT REVASCULARIZATION (%)	0
NEED FOR CARDIAC/VASC. OPERATION (%)	0
MAJOR VASCULAR COMPLICATIONS (%)	2.1
MAJOR BLEEDING (%)	1.0
ACUTE KIDNEY INJURY (%)	1.4
SEVERE HYPOTENSION (%)	8.9
CPR (%)	1.7
MACE (%)	3.1

Supplementary Table S1. In-hospitalclinical endpoints, n = 293. Data is exposed as percentages. CVE: cerebrovascular event. AMI: acute myocardial infarction. CPR: cardio-pulmonary resuscitation. MACE: major adverse cardiovascular events.

VARIABLE	BEFORE IVAC 2L	IVAC 2L ON	AFTER IVAC 2L
**HEART RATE (BPM)**	73.4±15.2	74.6±14.3	73.2±13
**CO (L/MIN)**	4.47±1.27*	4.78±1.29	4.65±1.18
**SBP (MMHG)**	117.3±23.7**	121.2±23	124.2±23.4
**DBP (MMHG)**	61.3±16.3**	63.8±14.9	64.6±16
**MAP (MMHG)**	80.2±17***	83±15.4	84.7±16.1
**MPCWP (MMHG)**	15.8±8	17.1±8.9	15.5±8
**CPO (WATTS)**	0.7±0.3**	0.8±0.3	0.9±0.3

Supplementary Table S3. Hemodynamic measurements taken before implementation of iVAC2L, during support and after interruption of support. Data is indicated as means±SD. P-values derive from t-tests or Wilcoxon's test for paired samples. * $p < 0.05$; ** $p < 0.01$; *** $p < 0.01$ compared to "iVAC 2L ON". Data availability on arterial blood pressure was 75%, and for CO, CPO and mPCWP it was 17%. CO: Cardiac Output. SBP: Systolic Blood Pressure. DBP: Diastolic Blood Pressure. MAP: Mean Arterial Pressure. mPCWP: Mean Pulmonary Artery Wedged Pressure. CPO: Cardiac Power Output.

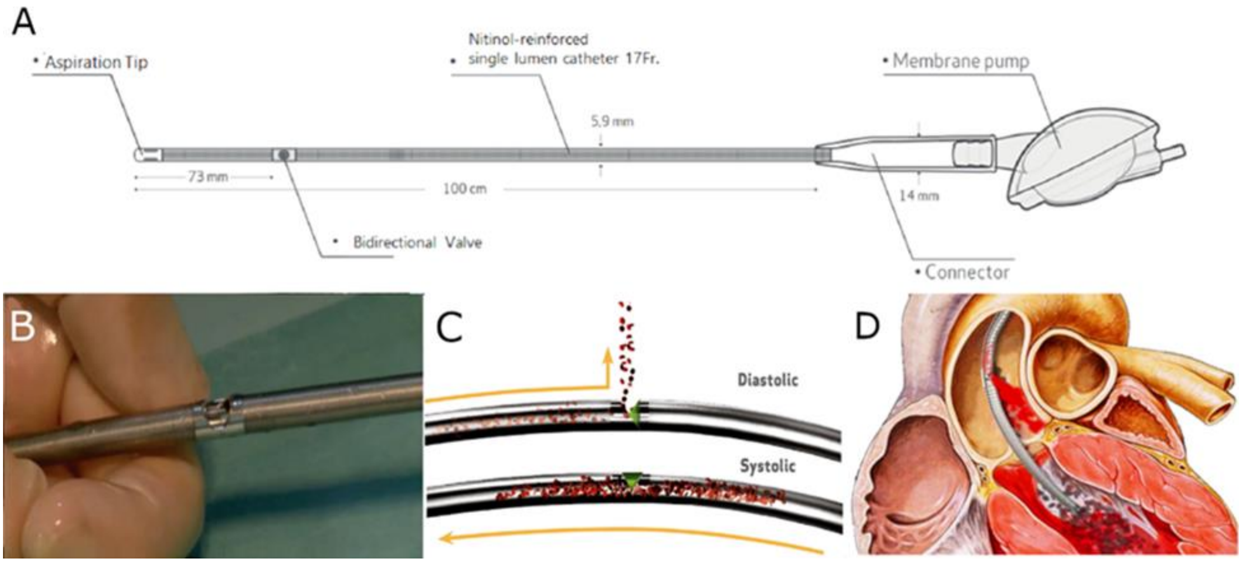

Supplementary Figure S1. (A) The iVAC2L concept. The catheter is inserted through the common femoral artery and positioned in the LV. The two-way valve is placed proximal to the aortic valve. (B) The two different configurations of the integrated two-way valve. In the closed position, blood flows from the inlet tip to the double chamber. In the open position, blood flows from the dual chamber to the ascending aorta. (C) Image of an experiment using ink to demonstrate the direction of the ejected jet during ejective inflation. (D) The iVAC2L catheter across the aortic valve. LV: Left Ventricle.

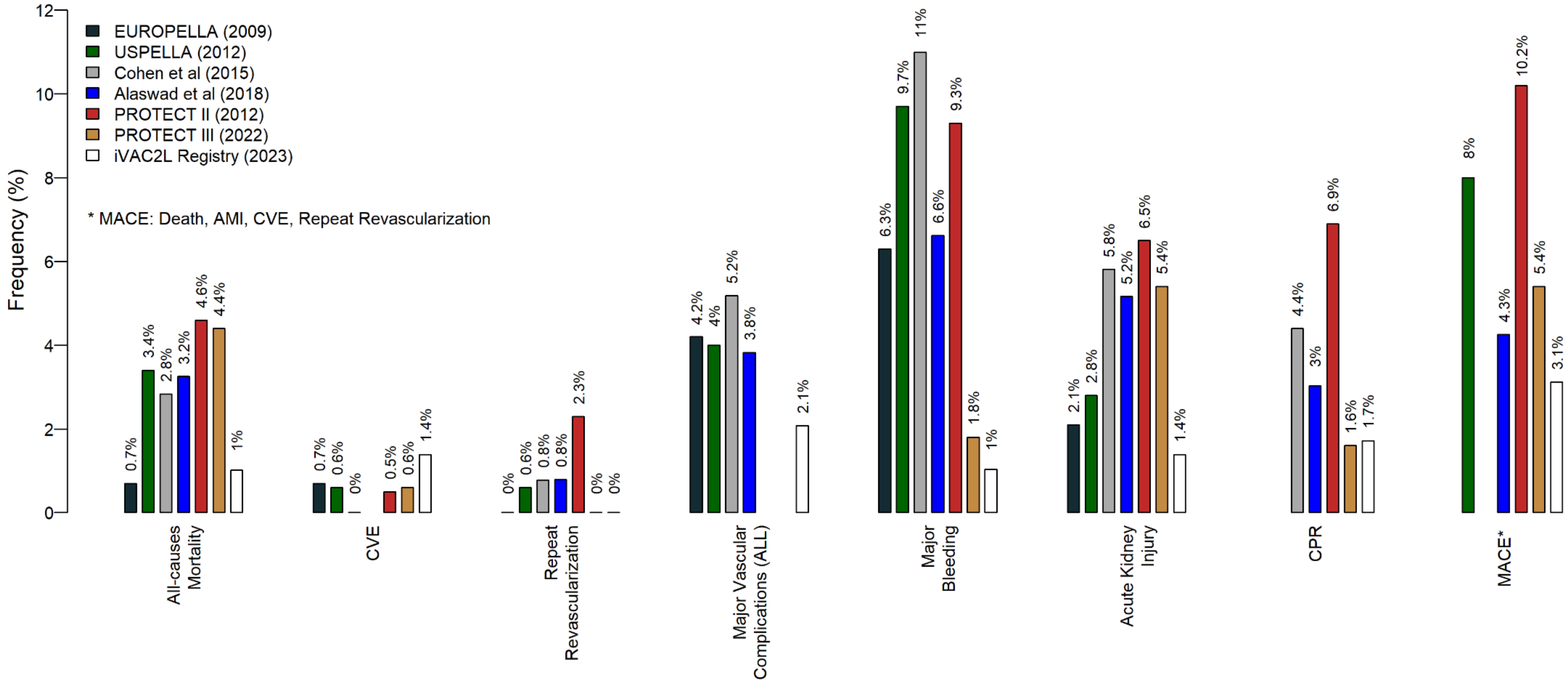

Supplementary Figure S2. Rates of in-hospital clinical outcomes in the iVAC2L Registry and the rates reported in other relevant studies in short-term mechanical circulatory support during high-risk PCI. CVE: Cerebrovascular Event. CPR: Cardiopulmonary Resuscitation. MACE: Major Adverse Cardiovascular Events. PCI: Percutaneous Coronary Intervention.
